# Cost-effectiveness of mass drug administration for control of scabies in Ethiopia: a decision-analytic model

**DOI:** 10.3389/frhs.2024.1279762

**Published:** 2024-09-18

**Authors:** Natalia Hounsome, Robel Yirgu, Jo Middleton, Jackie A. Cassell, Abebaw Fekadu, Gail Davey

**Affiliations:** ^1^ Brighton and Sussex Centre for Global Health Research, Brighton and Sussex Medical School, University of Sussex, Brighton, United Kingdom; ^2^ Center for Innovative Drug Development and Therapeutic Trials for Africa (CDT-Africa), Addis Ababa University, Addis Ababa, Ethiopia; ^3^ Department of Primary Care and Public Health, Brighton and Sussex Medical School, University of Sussex, Brighton, United Kingdom; ^4^ School of Public Health, Addis Ababa University, Addis Ababa, Ethiopia

**Keywords:** scabies, mass drug administration, Ethiopia, cost-effectiveness, ivermectin

## Abstract

**Background:**

The strategies to control scabies in highly endemic populations include individual case/household management and mass drug administration (MDA). We used a decision-analytic model to compare ivermectin-based MDA and individual case/household management (referred to as “usual care”) for control of scabies in Ethiopia at different prevalence thresholds for commencing MDA.

**Methods:**

A decision-analytic model was based on a repeated population survey conducted in Northern Ethiopia in 2018–2020, which aimed to evaluate the secondary impact of single-dose ivermectin MDA for the control of onchocerciasis on scabies prevalence. The model estimates the number of scabies cases and costs of two treatment strategies (MDA and usual care) based on their effectiveness, population size, scabies prevalence, compliance with MDA, medication cost, and other parameters.

**Results:**

In the base-case analysis with a population of 100,000 and scabies prevalence of 15%, the MDA strategy was both more effective and less costly than usual care. The probability of MDA being cost-effective at the current cost-effectiveness threshold (equivalent to the cost of usual care) was 85%. One-way sensitivity analyses showed that the MDA strategy remained dominant (less costly and more effective) in 22 out of 26 scenarios. MDA was not cost-effective at scabies prevalence <10%, MDA effectiveness <85% and population size <5,000. An increase in the cost of ivermectin from 0 (donated) to 0.54 US$/dose resulted in a decrease in the probability of MDA being cost-effective from 85% to 17%. At 0.25 US$/dose, the MDA strategy was no longer cost-effective.

**Conclusions:**

The model provides robust estimates of the costs and outcomes of MDA and usual care and can be used by decision-makers for planning and implementing scabies control programmes. Results of our analysis suggest that single-dose ivermectin MDA is cost-effective in scabies control and can be initiated at a scabies prevalence >10%.

## Background

Scabies is a neglected tropical disease (NTD) caused by the ectoparasite *Sarcoptes scabiei.* According to the estimations of the World Health Organisation, scabies affects more than 200 million people at any time. Prevalence estimates range from 0.2% to 71%, with the highest rates reported in resource-poor countries (1). The estimated global burden of scabies per 100,000 people is 71.11 DALYs (Disability-Adjusted Life Years) (2). Scabies transmission generally occurs by protracted skin-to-skin contact with infected individuals. In cases with high rates of mite burden (such as crusted scabies), transmission can also occur via infested fomites (clothing, bedding, etc.) (3). For most people who have not previously had scabies, an asymptomatic incubation period (up to 6 weeks) is followed by a reaction to the mites and their products, which causes intense itch, affecting sleep and quality of life. Infestation can be complicated by bacterial skin infections, including impetigo and abscess, and lead to septicaemia and rheumatic heart disease (4).

Scabies is common in sub-Saharan countries, including Ethiopia, Liberia, Nigeria, Cameroon, Malawi and Ghana (5). In Ethiopia, Amhara, Tigray and Oromia regions carry the main burden of scabies (6). In 2015 there was a major scabies outbreak in north Ethiopia caused by draught. In the Amhara region, the prevalence of scabies ranged from 2% to 67% in different districts (7). Children were disproportionally affected by scabies compared to adolescents and adults. 49% of cases were school-aged children, and 30% had secondary bacterial infections (7). In the West Gojjam Zone (2017), the prevalence of scabies among religious school students was 35% (8). A systematic review and meta-analysis of studies on scabies prevalence in Ethiopia conducted in 2019 estimated an overall prevalence of 14.5% (9).

In response to the 2015 scabies outbreak, the Federal Ministry of Health in Ethiopia developed a Scabies Outbreak Preparedness and Response Plan (6). The Plan included scabies surveillance and outbreak investigation; case management, awareness raising and community mobilisation; staff training; improving water supply, sanitation and hygiene (WASH services); advocacy and resource mobilisation; and supply chain management.

Scabies control included mass treatment of people in the affected areas with a prevalence >15%, including infested persons, their contacts and all other community members (except children < 2 years old, pregnant women and lactating mothers). In areas with prevalence < 15%, individual cases and close contacts (e.g., family members and sexual contacts) had to be treated. Scabies treatment included ivermectin tablets, permethrin cream, benzyl benzoate emulsion, and sulphur cream/ointment (6). Populations at risk (in schools, prisons, care centres, and childhood institutions) had to be closely monitored for scabies infestations. However, the prevalence of scabies remained high in 2018 in the Gondar area, varying from 15% in North Gondar to 39.2% in Central Gondar (10).

In 2019, the WHO conducted an informal consultation meeting on a Framework for Scabies Control aiming to find an agreement on common strategies for the global control of scabies (11). The resulting document was published in 2021 and included recommendations for mapping disease burden, delivering interventions, and establishing an appropriate monitoring and evaluation framework (12). According to the Framework, mass drug administration (MDA) is recommended in areas with a community prevalence of 10% or higher. This prevalence threshold was set informally through expert consultation (“consensus threshold”). The MDA regimen includes two doses of oral ivermectin (200 μg/kg) given 7–14 days apart. Ivermectin is currently not approved for pregnant women, lactating mothers in the first week, and children weighing *<*15 kg or *<*90 cm in height. Topical treatments are recommended for these populations, including permethrin or benzyl benzoate, where permethrin is unavailable. 3–5 rounds of annual MDA are required, with a minimum coverage of 80% of the population. The suggested threshold for terminating MDA is a community prevalence of <2% (12).

Following the recognition of scabies as a neglected tropical disease by WHO, it was included in the WHO NTD Roadmap 2021–2030, which sets strategies for the development of guidance on the implementation of preventive chemotherapy and integrating scabies control with other NTD programs where ivermectin is already used (e.g., onchocerciasis and lymphatic filariasis). In Ethiopia, the Federal Ministry of Health implements onchocerciasis and lymphatic filariasis MDA programmes as a part of the National Neglected Tropical Diseases Master Plan launched in 2013 (13). Amhara is among the endemic regions where biannual onchocerciasis MDA is implemented (14).

Additional evidence should be generated to inform decision-makers about allocating resources to integrate scabies control into existing MDA programmes. While onchocerciasis MDA includes one dose of oral ivermectin (150 μg/kg) administered every six months, the recommended regimen for scabies control assumes two doses of ivermectin (200 μg/kg) given 7–14 days apart. There is a discrepancy between the prevalence thresholds for commencing MDA for scabies: >10% recommended by the WHO Framework (12) and ≥15% currently accepted in Ethiopia (6). There are also questions regarding the cost of ivermectin (presently donated) and whether the MDA programme would be cost-effective when ivermectin has to be purchased.

To address these questions, we developed a decision-analytic model to evaluate the cost-effectiveness of ivermectin-based MDA in populations with scabies. The model was populated with data from a repeated population survey study in Ayu Guagusa district, Amhara regional state, Northern Ethiopia, in 2018–2020 (15), which evaluated the impact of the single-dose ivermectin-based MDA for the control of onchocerciasis on the prevalence of scabies. The model predicts the number of scabies cases, medication costs, and the probability of MDA being cost-effective at a specified cost-effectiveness threshold. The model allows for changing population size, prevalence of scabies, compliance with MDA, medication cost, treatment effectiveness, and other parameters. The model aims to assist decision-makers in planning and integrating scabies control programmes with existing NTD programmes.

## Methods

### Study design and setting

A static decision-analytic model was developed in Microsoft Excel 2010 to compare two strategies for controlling scabies in endemic areas: individual case/household management, referred to as “usual care,” and MDA. The model was developed using the recommended methods (16–18) and reported according to Consolidated Health Economic Evaluation Reporting Standards (CHEERS) (19).

**Figure 1:**
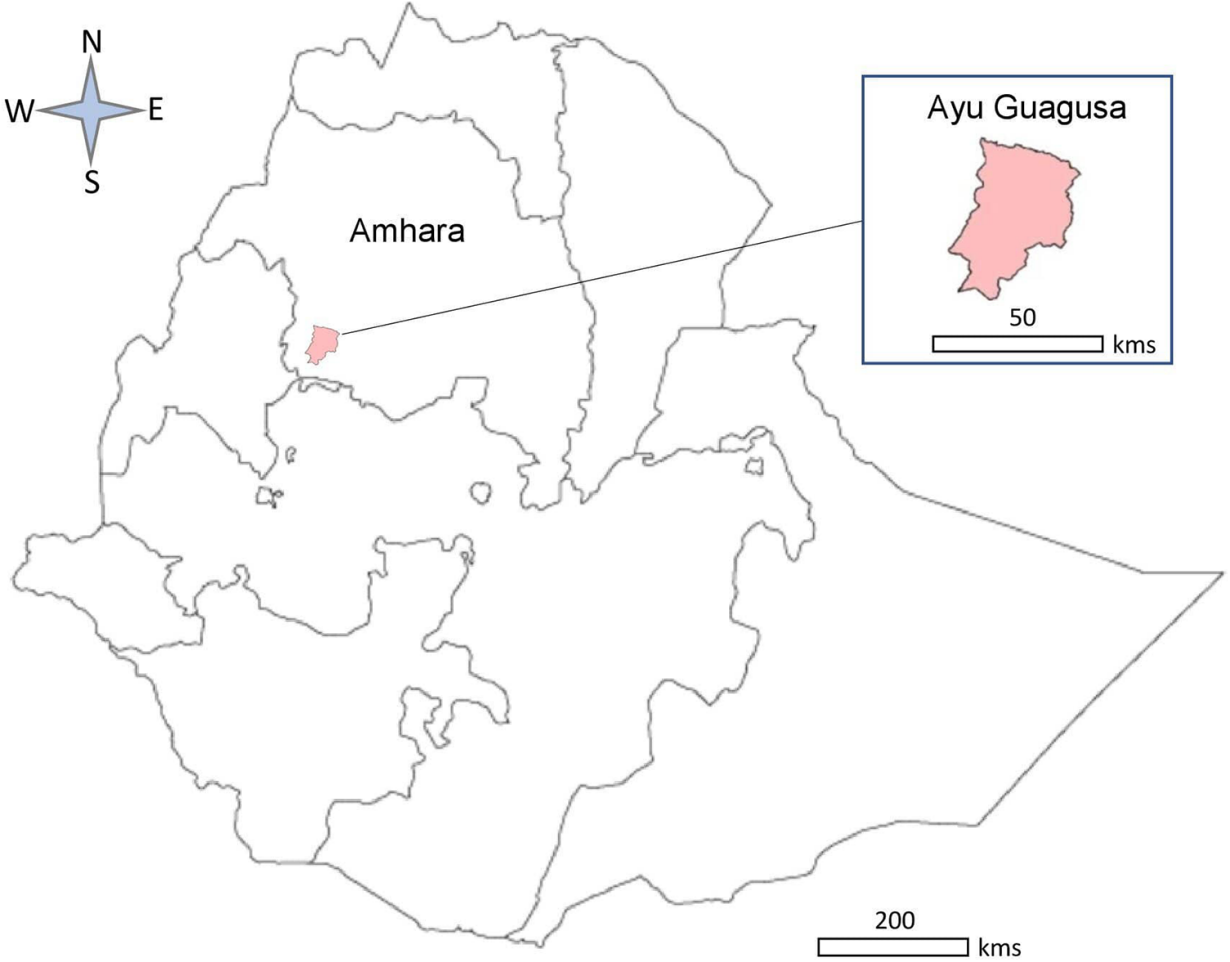
Map of Ethiopia showing Ayu Guagusa district.

The model was based on a repeated population survey conducted in Ayu Guagusa district, Amhara regional state, Northern Ethiopia, in 2018. The study aimed to evaluate the secondary impact on scabies prevalence of single-dose ivermectin-based MDA for control onchocerciasis in Northern Ethiopia. A detailed description of this study is published elsewhere (15). Briefly, Ayu Guagusa district (Figure 1) was purposively selected among 11 other districts in the Awi zone of the Amhara region where MDA to eliminate onchocerciasis was underway. Six out of 21 *kebeles* (the lowest administrative unit comprising 3,000–5,000 people) were selected using random sampling, and one *gote* (small village including 20–30 households) from each *kebele* was surveyed. The description of sampling and sample size calculation is presented in (15). The collected data included sociodemographic characteristics, household size, scabies symptoms (each, typical lesions), contact history with a person showing scabies-suggestive symptoms, taking ivermectin tablets during the last round of MDA against onchocerciasis and other characteristics. Data were collected using purpose-designed questionnaires translated into the local language (Amharic) and piloted with 12 people selected from a *kebele* near the study district. Informed consent was obtained from the adult study participants and parents of children <15 years old, and assent from minors 15–17 years old in addition to parents’ or guardians’ consent. Clinical examination was carried out by four nurses and one health officer trained in diagnosing scabies. The diagnosed individuals were referred to a healthcare facility (Health Centre or Health Post) to receive treatment for scabies. Data from the paper-based questionnaires were entered in an electronic template prepared using Epidata V.3.01 (EpiData Association, Odense).

To inform the model, a short survey was conducted with 15 healthcare professionals responsible for managing people with scabies at Health centres/posts in the affected areas in the Ayu Guagusa district. The questionnaire asked about the treatments provided to patients with scabies, pregnant women, children <5 years, and people with crusted scabies. Questions were also asked about the return visits with symptoms of scabies and treatments provided.

### Model description

**Figure 2:**
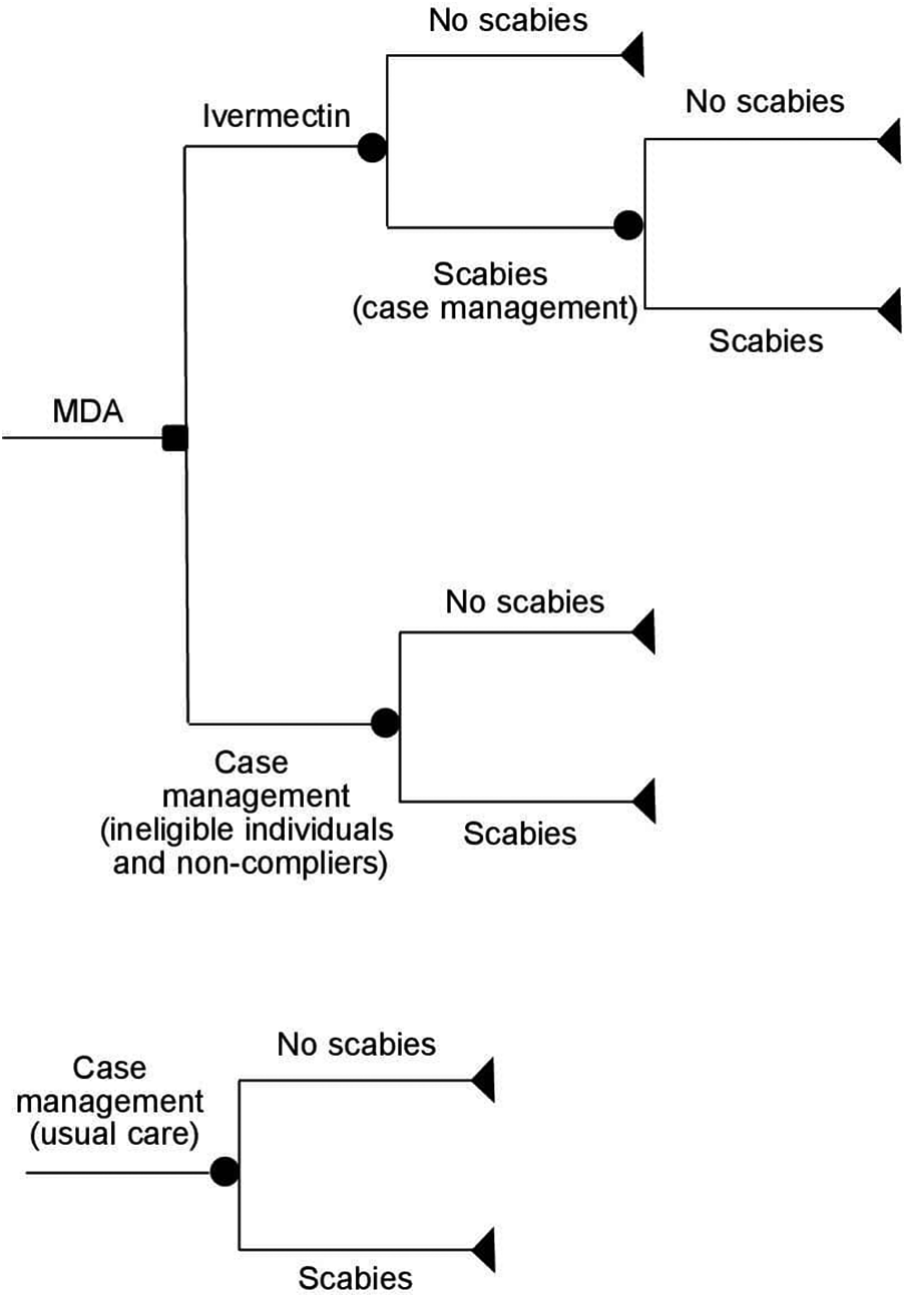
Schematic representation of the decision-analytic model.

The decision trees used in the model are shown in Figure 2. The list of model parameters and assumptions used in the model are provided in Supplementary Appendix 1 and Supplementary Appendix 2. The model assumes that all household members in the outbreak area were offered one dose of ivermectin MDA (150 μg/kg). This excludes pregnant/lactating women and children <5 years of age who were not eligible for the ivermectin treatment (14). Ineligible individuals with scabies symptoms and those who were eligible for MDA but did not take it (non-compliers) were offered topical treatment, which may include one dose of permethrin 5% cream, sulfur cream/ointment 5% or 10% or benzyl benzoate 20% cream (usual care). Individuals with crusted scabies and secondary infections also received antibiotics (amoxicillin 250/500 mg, cloxacillin 250/500 mg, or doxycycline 100/200 mg). As a part of usual care, a proportion of patients with scabies had their contacts/household members treated with either ivermectin or topical treatment. The number of contacts treated was assumed to be the same as the mean number of people in a household (4.64, Supplementary Appendix 1). The model assumes that people who still have scabies after the initial treatment (ivermectin or topical treatment) received an additional treatment consisting of one dose of permethrin or benzyl benzoate. The model does not include hospital costs since no hospitalisations due to scabies or complications from scabies were reported in this study. The model assumes a 6-month time horizon consistent with biannual MDA administration. For more model assumptions, see Supplementary Appendix 1.

### Population

The model population resembles the survey cohort which included household members in six *gotes*. The average number of people in a household and the proportion of children <5 years of age were derived from the survey. In the absence of survey data on pregnant/lactating women, we used estimates provided by the Federal Ministry of Health (6). In the base case scenario, a district population of 100,000 was assumed, as per Federal Ministry of Health Preparedness and Response Plan (6). In sensitivity analyses, the population varied from 5,000 to 200,000 people. The proportions of pregnant/lactating women and children <5 years of age were assigned to be the same in all simulations.

### Probabilities

The list of probabilities used in the model is shown in Supplementary Appendix 2, and the list of assumptions in Supplementary Appendix 1. In the base-case analysis, the probabilities of taking ivermectin for MDA, as well as the probabilities of developing scabies, were derived from the population survey (15). In sensitivity analysis, all eligible populations, excluding children <5 years and pregnant/lactating women, were assumed to take ivermectin. The probability of receiving usual care was considered to be equal to scabies prevalence, given that in actual life, prevalence estimates are based on diagnosed cases. In the base-case analysis, the prevalence of scabies was set at 15%. In the sensitivity analysis, it varied from 1% to 25%. The probability of having scabies after MDA was derived from the population survey at a 6-month follow-up. The probability of having scabies after usual care was derived for participants who did not take ivermectin for MDA and developed scabies. The probabilities of treating contacts of people with scabies, the probabilities of repeat visits with scabies and the probabilities of prescribing different topical treatments were derived from the survey. An equal probability of being treated with different antibiotics was assumed (Supplementary Appendix 1).

### Costs

Supplementary Appendix 2 shows the list of costs used in the model. Costs associated with MDA included medication and MDA training for healthcare professionals. The latter was based on the reimbursement of travel and subsistence expenses for trainees and trainers attending the MDA training events.

The medication costs included ivermectin (150 μg/kg); permethrin 5% cream; sulfur cream/ointment 5% or 10%; benzyl benzoate 20% cream; and antibiotics (joint probability of prescribing amoxicillin 250/500 mg, cloxacillin 250/500 mg and doxycycline 100/200 mg).

In the base-case scenario, ivermectin was assumed to be donated to the MDA programme. Alternative costing scenarios were based on: (i) an estimate of the opportunity cost for donated ivermectin, 1.51 US$ per three tablets (20), and (ii) a negotiated price for the ivermectin MDA programme in Fiji, 0.18 US$ per 3 mg tablet (21). The model assumes a single dose of ivermectin equivalent to three 3 mg tablets of ivermectin for a 60 kg person.

The MDA distribution costs were not included in this study, given that they were integrated into the ongoing onchocerciasis MDA programme and, therefore, difficult to estimate. However, the model allows distribution costs to be included when newly established programmes are considered.

The reimbursement costs for healthcare professionals and community health workers attending the MDA training were obtained retrospectively. The training included a Consultative meeting with zonal and district administrators and NTD experts and an Orientation session for Health Extension Workers about field operations during the MDA.

The cost of usual care included the cost of topical treatment, the cost of treating contacts, the cost of antibiotics for crusted scabies and secondary infections, and the cost of repeat treatment.

The cost of the MDA strategy included the cost of ivermectin, the cost of MDA training, and the cost of usual care for ineligible individuals, non-compliant individuals, and those who remained with scabies after MDA.

### Health outcomes

The effectiveness outcome in the model was the number of clinical scabies cases. Given that treatment decisions are based on symptoms in a real-world situation, the model does not account for infected individuals who did not have clinical signs.

### Discounting

Discounting was not applied since the model assumes a 6-month time horizon.

### Cost-effectiveness analysis

The incremental cost-effectiveness ratio (ICER) was defined as a difference in cost between MDA and usual care divided by a minus difference in the number of scabies cases between MDA and usual care: ICER = ΔCost/–ΔEffectiveness. The denominator was multiplied by −1 to reflect that a positive outcome was defined as a decrease in the number of scabies cases (scabies cases avoided).

One-way sensitivity analyses were conducted using the lower and the upper limit values for each model parameter (Supplementary Appendix 2) to demonstrate the sensitivity of the model to one-at-a-time changes in model parameters. Probabilistic sensitivity analysis was conducted using 1,000 Monte-Carlo simulations to test the sensitivity of the model to simultaneous changes in all parameters. The probability of MDA being cost-effective was calculated at the cost-effectiveness threshold equivalent to the cost of usual care.

## Results

### MDA strategy

According to the population survey, 85.6% of participants (1,464 out of 1,711) had taken ivermectin in the past six months. Ivermectin was administered as a single dose of 150 µg/kg, excluding pregnant/lactating women and children <5 years of age. The proportion of children <5 years of age in the population sample was 9.5%. The estimated proportion of pregnant/lactating women was 2.9%. The latter included women 15–50 years of age who did not take ivermectin. The sensitivity analysis also included an earlier estimate of 3.5% by the Federal Ministry of Health (6). The estimated proportion of non-compliers (those eligible for MDA but did not take ivermectin) was 2%.

Assuming donated ivermectin, the estimated cost of treatment, including MDA training, was 0.01 US$/person for those who were scabies-free after taking ivermectin, 3.13 US$/person for those who still had clinical scabies after taking ivermectin and received topical treatments; and 3.12 US$ for ineligible individuals and non-compliers who received usual care. Sensitivity analysis also considered ivermectin costs of 0.54 US$/dose (21) and 1.51 US$/dose (20).

### Usual care

According to data from 15 healthcare professionals involved in managing patients with scabies, permethrin was the first treatment of choice for usual care (14 respondents). Three healthcare professionals also mentioned benzyl benzoate and sulphur cream as a first-line treatment. One respondent reported prescribing ivermectin as a part of usual care. Patients with crusted scabies were also given antibiotics (amoxicillin, cloxacillin, or doxycycline). Repeat treatments included permethrin and benzyl benzoate. 20% of patients were given medication to treat their contacts. No hospitalisations due to scabies or its complications were reported in this study.

The estimated proportion of pregnant/lactating women among scabies patients was 8.0%, children <5 years 20.7%, and patients with crusted scabies 2.7%. Repeat visits with scabies were reported for 5.8% of patients. The average medication cost was 2.92 US$/person for scabies-free patients after the initial treatment and 3.12 US$/person for the repeat treatment. The cost of usual care did not include costs of appointments with healthcare professionals and travel expenses, since these were not collected in the study.

### Cost-effectiveness analysis

The base-case cost-effectiveness analysis was conducted for a population of 100,000 and a scabies prevalence of 15% (the recommended threshold for the initiation of MDA) (6). The full list of model parameters is shown in Supplementary Appendix 2. The results of the base case and sensitivity analyses are presented in Table 1. In the base case scenario, the number of scabies cases was 1,583 for MDA vs. 2,130 for usual care, and the total cost was 33,490 US$ for MDA vs. 44,176 US$ for usual care. The MDA strategy was both more effective and less costly compared to usual care. The probability of MDA being cost-effective at the current cost-effectiveness threshold (cost of usual care) was 85%.

**Table 1:** Results of the base-case and deterministic sensitivity analysis of MDA compared to usual care.

Parameter	MDA	Usual care	Difference	ICER^a^	Probability cost-effective (%)
Base-case analysis					
Number of scabies cases	1,583	2,130	−547		
Cost	33,491	44,176	−10,685	Dominant	85%
Sensitivity analysis					
Scabies prevalence 10%					
Number of scabies cases	1,481	1,420	61		
Cost	31,370	29,450	1,920	Dominated	N/A
Scabies prevalence 33.5%					
Number of scabies cases	1,961	4,757	−2,796		
Cost	41,336	98,659	−57,323	Dominant	100%
Population size 5,000					
Number of scabies cases	79	107	−27		
Cost	2,301	2,209	92	3.36	54%
Population size 200,000					
Number of scabies cases	3,166	4,260	−1,094		
Cost	66,322	88,351	−22,030	Dominant	85%
Population taking MDA 65.3%					
Number of scabies cases	1,713	2,130	−417		
Cost	35,628	44,176	−8,548	Dominant	79%
Population taking MDA 86.7%					
Number of scabies cases	1,576	2,130	−554		
Cost	33,353	44,176	−10,823	Dominant	86%
No scabies after MDA 75.0%					
Number of scabies cases	3,346	2,130	1,216		
Cost	70,044	44,176	25,869	Dominated	N/A
No scabies after MDA 94.0%					
Number of scabies cases	1,036	2,130	−1,094		
Cost	22,146	44,176	−22,030	Dominant	100%
No scabies after usual care 85.0%					
Number of scabies cases	1,672	2,250	−578		
Cost	33,509	44,200	−10,692	Dominant	83%
No scabies after usual care 97.8%					
Number of scabies cases	245	330	−85		
Cost	33,215	43,805	−10,590	Dominant	85%
Population required repeat treatment 1%					
Number of scabies cases	1,583	2,130	−547		
Cost	33,221	43,813	−10,592	Dominant	85%
Population required repeat treatment 50.0%					
Number of scabies cases	1,583	2,130	−547		
Cost	35,996	47,547	−11,551	Dominant	85%
Population prescribed antibiotics 26.7%					
Number of scabies cases	1,583	2,130	−547		
Cost	32,614	42,997	−10,382	Dominant	85%
Population prescribed antibiotics 40.0%					
Number of scabies cases	1,583	2,130	−547		
Cost	34,367	45,355	−10,988	Dominant	85%
Cost of ivermectin 0.54 US$					
Number of scabies cases	1,583	2,130	−547		
Cost	69,104	44,446	24,659	45.1	17%
Cost of ivermectin 1.51 US$					
Number of scabies cases	1,583	2,130	−547		
Cost	132,774	44,928	87,845	160.6	1%
Cost of permethrin 2.00 US$					
Number of scabies cases	1,583	2,130	−547		
Cost	28,562	37,544	−8,982	Dominant	83%
Cost of permethrin 3.00 US$					
Number of scabies cases	1,583	2,130	−547		
Cost	38,419	50,807	−12,388	Dominant	85%
Cost of sulphur cream 2.00 US$					
Number of scabies cases	1,583	2,130	−547		
Cost	33,283	43,897	−10,614	Dominant	85%
Cost of sulphur cream 2.50 US$					
Number of scabies cases	1,583	2,130	−547		
Cost	33,698	44,455	−10,757	Dominant	85%
Cost of benzyl benzoate 1.00 US$					
Number of scabies cases	1,583	2,130	−547		
Cost	32,844	43,306	−10,462	Dominant	85%
Cost of benzyl benzoate 2.00 US$					
Number of scabies cases	1,583	2,130	−547		
Cost	34,137	45,046	−10,909	Dominant	85%
Cost of antibiotics (amoxicillin, cloxacillin, or doxycycline) 0.70 US$					
Number of scabies cases	1,583	2,130	−547		
Cost	31,730	41,807	−10,077	Dominant	86%
Cost of antibiotics (amoxicillin, cloxacillin, or doxycycline) 1.90 US$					
Number of scabies cases	1,583	2,130	−547		
Cost	36,225	47,856	−11,630	Dominant	85%
Cost of MDA training 434 US$					
Number of scabies cases	1,583	2,130	−547		
Cost	33,203	44,176	−10,973	Dominant	86%
Cost of MDA training 770 US$					
Number of scabies cases	1,583	2,130	−547		
Cost	33,491	44,176	−10,685	Dominant	85%

^a^ ICER = ΔCost/ - ΔNumber of scabies cases; N/A, not applicable.

One-way sensitivity analyses (Table 1) show that the MDA strategy remained dominant (less costly and more effective) in 22 of 26 scenarios. In two scenarios the MDA strategy was dominated (more costly and less effective). These include scenarios with scabies prevalence ≤10% and MDA effectiveness ≤72%. An increase in the ivermectin cost from 0 to 0.54 US$/dose resulted in a decrease in the probability of MDA being cost-effective from 85% to 17%. At 0.54 US$/dose, the MDA strategy was not cost-effective, since the ICER (45.1 US$) was above the cost-effectiveness threshold (3.12 US$) for the scabies case avoided. For a population of 100,000, the MDA costs varied from 10,928 US$ to 132,774 US$, depending on ivermectin cost, MDA effectiveness, and other parameters (see Table 1).

**Figure 3:**
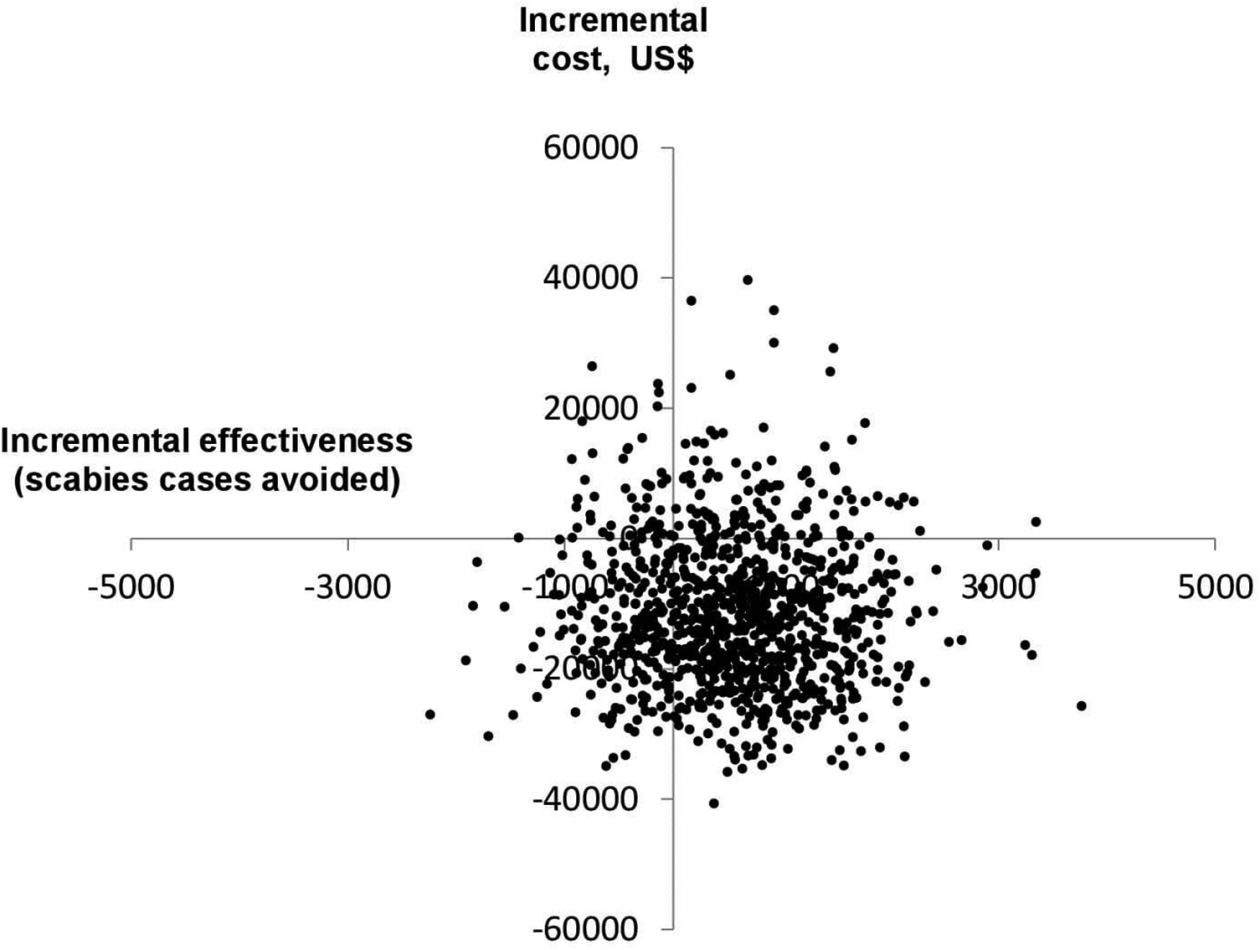
Cost-effectiveness plane for the base-case scenario, generated using 1,000 Monte-Carlo simulations.

Figure 3 shows the results of the probabilistic sensitivity analysis conducted using 1,000 Monte-Carlo simulations for the base-case scenario (population of 100,000, scabies prevalence of 15% and donated ivermectin). Most ICER estimates fell into the lower right quadrant of the cost-effectiveness plane, where the MDA strategy is less costly and more effective than usual care (MDA dominant).

**Figure 4:**
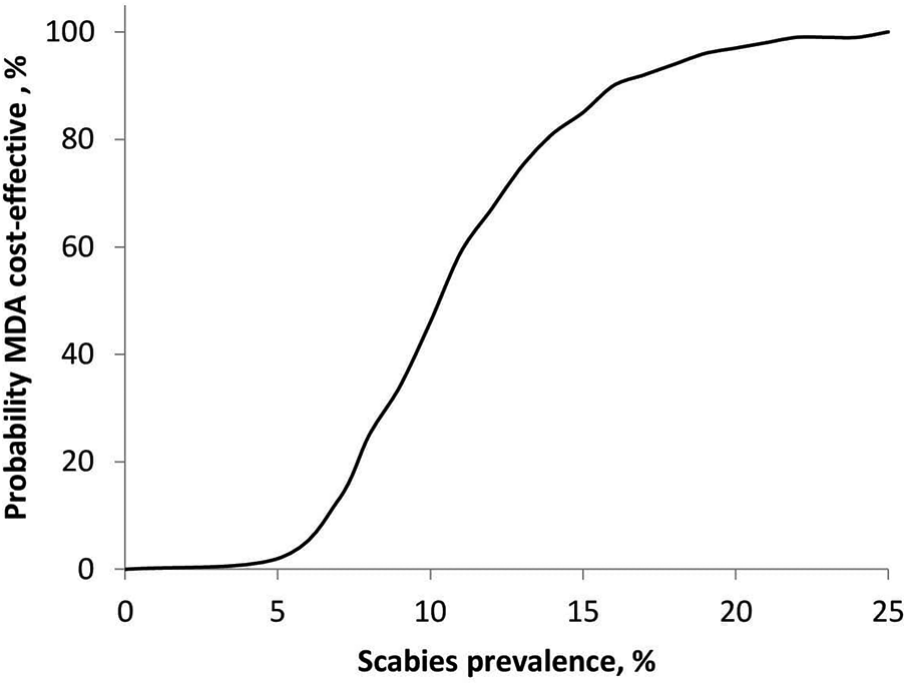
Probability of MDA being cost-effective as a function of scabies prevalence.

Figure 4 shows the relationship between the probability of MDA being cost-effective and scabies prevalence for a population of 100,000. The MDA strategy was not cost-effective at scabies prevalence ≤10%, where the probability of MDA being cost-effective was below 50%. The probability of MDA being cost-effective was 100% at a population prevalence above 23%.

**Figure 5:**
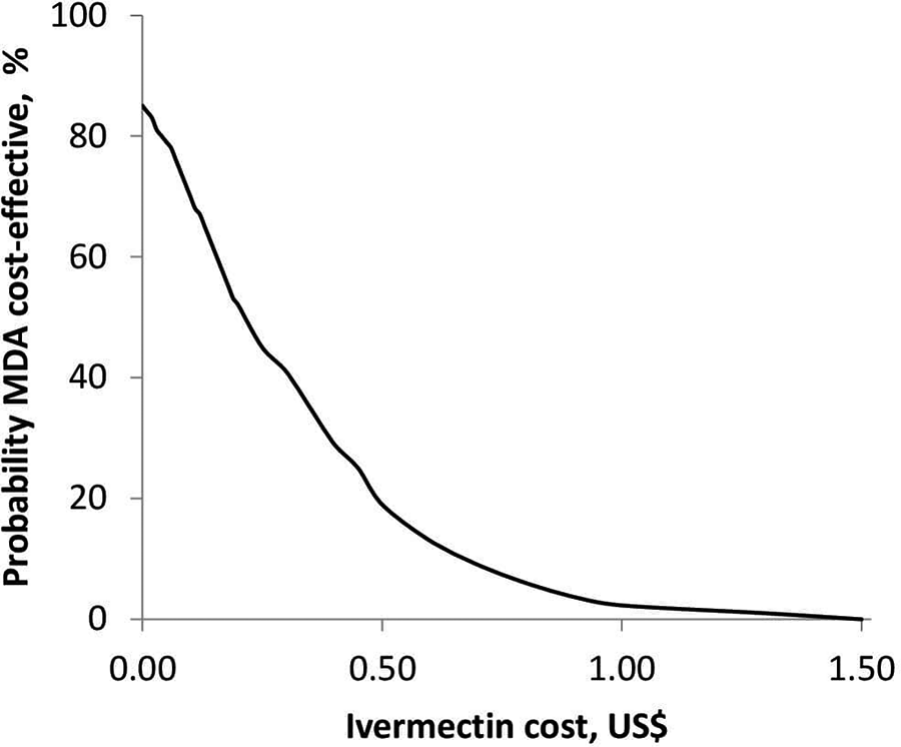
Probability of MDA being cost-effective as a function of ivermectin cost.

Figure 5 shows the relationship between the probability of MDA being cost-effective and the cost of ivermectin at a scabies prevalence of 15% and a population of 100,000. The probability of MDA being cost-effective was 85% when ivermectin was provided for free. An increase in the cost of ivermectin resulted in a decrease in the probability of MDA being cost-effective. The MDA strategy was no longer cost-effective at an ivermectin cost above 0.25 US$ per dose.

**Figure 6:**
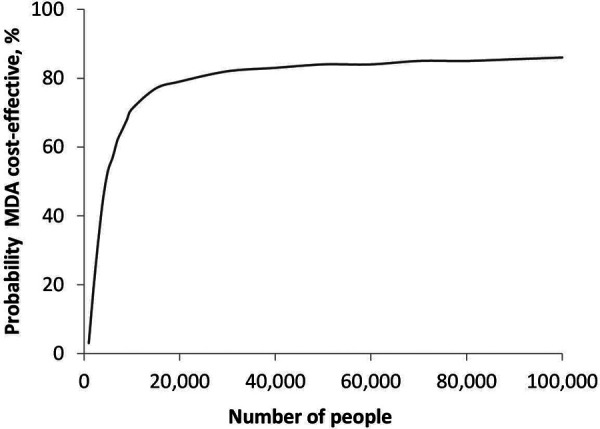
Probability of MDA being cost-effective as a function of population size.

Figure 6 shows the relationship between the probability of MDA being cost-effective and population size at a scabies prevalence of 15%. In populations below 5,000 people, the MDA strategy was not cost-effective. The probability of MDA being cost-effective was 85% for a population of 100,000. A further increase in the population size to 200,000 resulted in an 86% probability of the MDA strategy being cost-effective.

## Discussion

This paper presents a robust decision-analytic model for estimating costs and effectiveness outcomes of ivermectin-based MDA compared to individual case/household management of scabies. In Ethiopia, scabies is co-endemic with onchocerciasis and lymphatic filariasis, and ivermectin-based onchocerciasis and lymphatic filariasis MDA programmes are currently implemented in these areas (13). Integrating scabies MDA into existing programmes is challenging. The recommended ivermectin regimen for scabies includes two doses of oral ivermectin (200 mg./kg) given 7 to 14 days apart (12), while onchocerciasis MDA comprises one dose (150 μg/kg) administered every six months (14). Our study demonstrates that a single-dose ivermectin MDA can be cost-effective in reducing scabies prevalence. At the time of writing this paper, we did not have empirical data on the effectiveness of the double-dose ivermectin MDA for scabies in Ethiopia. Should such data emerge in the future, the proposed model can be used to estimate the cost-effectiveness of the double-dose MDA.

Several economic models have been developed to predict the effectiveness and cost-effectiveness of scabies interventions. The model by Bachewar et al. (2009) was based on a clinical trial comparing the safety, efficacy, and cost-effectiveness of benzyl benzoate, permethrin, and ivermectin in individual patients with scabies attending Nagpur Hospital, India (22). This was a decision-tree-based Markov cohort model to estimate costs and cure rates of different treatment regimens over a two-week time horizon. The model suggested that a single dose of 200 μg/kg ivermectin was the most effective treatment for scabies (22).

The model developed by Gilmore (2011) (23) was a network-dependent Monte-Carlo transmission model to predict the effects of various treatment strategies on scabies prevalence in child populations. The model did not compare any specific treatment regimens but focused on the density and frequency of treatments. The model suggested that mass screening and treatment of all affected individuals at regular intervals is the most appropriate strategy (23).

The compartmental model developed by Kinyanjui et al. (2018) (24) was based on Markov chain Monte Carlo simulations to predict costs and outcomes of scabies treatments in residential care homes for elderly people. The model followed the natural course of scabies (Susceptible-Exposed-Infectious framework) to predict the numbers of infectious individuals and the costs of treatment with a single course of permethrin (two applications a week apart) or two doses of oral ivermectin (two weeks apart) (24).

The compartmental model developed by Lydeamore et al. (2019) (23) aimed to capture the natural history of the mite's life cycle in relation to the host (susceptible - infectious - infectious and with eggs present - having only eggs). The model was used to compare MDA with presumed ovicidal (e.g., permethrin and benzyl benzoate) vs. non-ovicidal (e.g., ivermectin) treatments. The results of this study suggest that at least two ivermectin treatments administered two weeks apart would be required to clear scabies (23). It should be noted that the presumption that permethrin is ovicidal in and of itself has recently been challenged by experimental work by Bernigaud et al. (2020) (25). They found that pure permethrin was not ovicidal, but commercial formulations common in the Global North were, potentially, partly due to their vehicles.

In contrast to dynamic models (23, 24, 26), our model does not account for scabies transmission. Static models tend to underestimate the number of people requiring treatment since infected individuals remain asymptomatic for up to a month while transmitting the disease. It should be mentioned that in the real world, treatment decisions are based on the number of scabies cases (prevalence) rather than the number of infected individuals. We populated the model with effectiveness data from a community-based study that should capture the effects of scabies transmission, access to treatment, treatment acceptance and adherence to treatment on the prevalence of scabies. At the current threshold for the initiation of MDA (scabies prevalence of 15%) and free ivermectin, there was an 85% probability of single-dose ivermectin MDA being cost-effective in populations over 5,000 people. MDA is not recommended at scabies prevalence <2% (12). There are currently no evidence-based recommendations for the prevalence between 2% and 10%, and decisions need to be made about available and affordable treatments. Our model-based analysis suggests that MDA is unlikely to be cost-effective at scabies prevalence below 10%, which agrees with the Framework for Scabies Control (12).

The cost of ivermectin can be a barrier to scabies control due to the higher cost of two-dose regimens and the current absence of drug donation programmes for scabies in Ethiopia. In this study, ivermectin was donated for the onchocerciasis MDA programme. In a study of MDA costs in Fiji, the negotiated price of ivermectin was 0.18 US$ per 3 mg tablet (21). This is equivalent to 0.54 US$ per dose of ivermectin (150 μg/kg) used in our study. Our model shows that the MDA strategy is unlikely to be cost-effective at ivermectin costs above 0.25 US$/dose. Therefore, improving access to low-cost ivermectin is essential for the success of scabies control programmes.

## Limitations

Our study has the following limitations:

The proposed model is a static model based on scabies prevalence (symptomatic cases). This may be considered a limitation since the model does not account for scabies transmission and, therefore, can underestimate the cost of treatment. Given that transmission rates depend on many factors and are difficult to predict, changing the prevalence numbers in the model can address this limitation.

This model was based on a study conducted in Ethiopia and reflects the current practice of scabies management in this country. The estimates of cost and effectiveness outcomes reported in this study may not apply elsewhere.

The study was conducted from a healthcare provider perspective and does not include patients’ out-of-pocket expenses, the cost of work productivity loss, etc. However, when adding these costs, the MDA strategy will be even more cost-effective due to the lower number of scabies cases compared to usual care.

This study included the costs of medication and MDA training only. We did not include the costs of ivermectin distribution since this was delivered by the existing staff within the onchocerciasis control programme (12). The intervention cost will increase should additional staff be employed to implement MDA.

The model did not consider the costs and consequences of scabies complications since no complications were reported in this study. This omission is unlikely to bias the model since no serious adverse reactions were reported for ivermectin MDA (27).

## Conclusion

Our model provides robust estimates of scabies cases and treatment costs depending on the threshold for initiating MDA, population size, treatment effectiveness, ivermectin cost and other parameters. The model-based analysis demonstrates that single-dose ivermectin MDA was cost-effective in populations with scabies. At the current threshold for the initiation of MDA in Ethiopia (scabies prevalence of 15%) and donated ivermectin, there was an 85% probability of single-dose ivermectin MDA being cost-effective in populations over 5,000 people. Our study suggests that ivermectin-based MDA can be initiated at scabies prevalence >10%, which is in agreement with the “consensus threshold” proposed by the Framework for Scabies Control (12). The high sensitivity of model estimates to the ivermectin cost stresses the importance of access to low-cost ivermectin for the success of MDA. In conclusion, the proposed model is robust, versatile and user-friendly. It can be adapted to different ivermectin regimens to integrate scabies MDA into existing MDA programmes. The model can be helpful for decision-makers in planning and implementing scabies control interventions in Ethiopia.

## Data Availability

The raw data supporting the conclusions of this article will be made available by the authors, without undue reservation.
