# Supplementary material for: Cost-effectiveness of mass drug administration for control of scabies in Ethiopia: a decision-analytic model
Source: Front Health Serv. 2024 Sep 18;4:1279762. doi: 10.3389/frhs.2024.1279762 (PMC11445614; doi:10.3389/frhs.2024.1279762)
Supplement: Supplementary file 1 [file Table1.docx]

Appendix 1. List of model assumptions

| **Model characteristic** | **Assumption/rationale** | **Source** |
| --- | --- | --- |
| **District population** | 100,000 | [6] |
| **Proportion of pregnant/lactating women** | 3.5% | [6] |
| **Proportion of children <5 years of age** | 9.8 % | [current study] |
| **Number of repeat treatments** | one | assumption |
| **Number of contacts** | 4.64  (average number of people in a household) | [current study] |
| **Treatment regimens** |  |  |
| MDA | Ivermectin 150 µg/kg one dose (three 3mg tablets for a 60kg person) excluding pregnant/lactating women and children <5 years of age | [6] |
| Usual care first treatment | Permethrin 5% cream, one pack;  Sulphur ointment 10%, one pack;  Benzyl benzoate 20% cream, one pack;  Ivermectin single dose (three 3mg tablets) | [current study] |
| Usual care pregnant/lactating women | Permethrin 5% cream, one pack;  Sulphur ointment 10%, one pack;  Benzyl benzoate 20% cream, one pack | [current study] |
| Usual care children <5 years of age | Permethrin 5% cream, one pack;  Sulphur ointment 10%, one pack;  Benzyl benzoate 20% cream, one pack | [current study] |
| Usual care crusted scabies | Permethrin 5% cream, one pack;  Benzyl benzoate 20% cream, one pack;  Antibiotics, one pack (equal probability of prescribing doxycycline 100 or 200 mg, amoxicillin 250 or 500mg, and cloxacillin 250 or 500mg) | [current study] |
| Usual care repeat treatment | Permethrin 5% cream, one pack;  Benzyl benzoate 20% cream, one pack | [current study] |
| Usual care treatment of contacts | Permethrin 5% cream, one pack/contact;  Benzyl benzoate 20% cream, one pack/contact | [current study] |
| **MDA training** |  |  |
| Consultative training event with zonal and district administrators, and NTD experts | One event at a district level, 14-21 attendants | [current study] |
| Orientation session for community health workers | One event at a district level, 21-34 attendants | [current study] |
| **Willingness-to-pay threshold** | 3.12 US$/person  cost of usual care | [current study] |
