# Supplementary material for: Cost-effectiveness of mass drug administration for control of scabies in Ethiopia: a decision-analytic model
Source: Front Health Serv. 2024 Sep 18;4:1279762. doi: 10.3389/frhs.2024.1279762 (PMC11445614; doi:10.3389/frhs.2024.1279762)
Supplement: Supplementary file 2 [file Table2.docx]

Appendix 2. List of model parameters with sources

| **Parameter** | **Base case** | **Lower limit** | **Upper limit** |
| --- | --- | --- | --- |
| Population size | 100,000  average district population [6] | 5,000  assumption | 200,000  assumption |
| Average number of people in a household | 4.64  [current study] | 4.64  [current study] | 5.0  [6] |
| % children <5 years | 9.8  [current study] | 9.8  [current study] | 14.0  [28] |
| % of pregnant/lactating women | 3.5  [6] | 2.9  [current study] | 3.5  [6] |
| Scabies prevalence, % | 15.0  threshold for MDA initiation [6] | 10.0  assumption | 33.5  [7] |
| % population taking ivermectin for MDA | 85.6  [current study] | 65.3  [29] | 86.7  all eligible population excluding children <5 years and pregnant/lactating women [6] |
| % no clinical scabies after MDA | 89.5  [current study] | 75.0  [30] | 94.0  [31] |
| % no clinical scabies after usual care | 85.8  [current study] | 85.0  permethrin [27] | 97.8  permethrin [28] |
| % population with crusted scabies | 2.7  [current study] | 0.2  [32] | 24  [32] |
| % population with repeat visits after usual care | 5.8  [current study] | N/A | N/A |
| % population prescribed ivermectin one dose, usual care | 3.3  [current study] | N/A | N/A |
| % population prescribed permethrin 5% cream, usual care | 80.3  [current study] | N/A | N/A |
| % population prescribed sulphur cream 5% or 10%, usual care | 7.4  [current study] | N/A | N/A |
| % population prescribed benzyl benzoate 20% cream, usual care | 11.4  [current study] | N/A | N/A |
| % population with treated contacts, usual care | 7.2  [current study] | N/A | N/A |
| % population with treated contacts, repeat visit, usual care | 37.5  [current study] | N/A | N/A |
| % population prescribed permethrin 5% cream, repeat visit, usual care | 75.0  [current study] | N/A | N/A |
| % population prescribed benzyl benzoate 20% cream, repeat visit, usual care | 25.0  [current study] | N/A | N/A |
| % population prescribed antibiotics (joint probability of prescribing doxycycline, amoxicillin, cloxacillin) | 33.3  [current study] | 26.7  -20% assumption | 40.0  +20% assumption |
| **Costs, US$** |  |  |  |
| Ivermectin | 0.00  donated [current study] | 0.54  negotiated price [21] | 1.51  estimate of the opportunity cost [20] |
| Permethrin 5% cream | 2.50  [current study] | 2.00  [current study] | 3.00  [current study] |
| Sulphur cream 5% or 10% | 2.25  [current study] | 2.00  [current study] | 2.50  [current study] |
| Benzyl benzoate 20% cream | 1.5  [current study] | 1.00  [current study] | 2.00  [current study] |
| Antibiotics (doxycycline, amoxicillin, cloxacillin) | 1.17  [current study] | 0.9  [current study] | 1.9  [current study] |
| MDA training:  Event 1. Consultative meeting for zonal and district administrators (14-21 attendants)  Event 2. Orientation session for community health workers (21-34 attendants) | 770.00  Events 1 and 2, maximum attendance  [current study | 434.00  Events 1 and 2, minimum attendance [current study] | 770.00  events 1 and 2, maximum attendance  [current study] |
